# Circulating Plasma microRNAs can differentiate Human Sepsis and Systemic Inflammatory Response Syndrome (SIRS)

**DOI:** 10.1038/srep28006

**Published:** 2016-06-20

**Authors:** Stefano Caserta, Florian Kern, Jonathan Cohen, Stephen Drage, Sarah F. Newbury, Martin J. Llewelyn

**Affiliations:** 1 Brighton and Sussex Medical School, Falmer, East Sussex, BN1 9PS, United Kingdom; 2 Brighton and Sussex University Hospitals NHS Trust, Eastern Road, Brighton, BN2 5BE, United Kingdom

## Abstract

Systemic inflammation in humans may be triggered by infection, termed sepsis, or non-infective processes, termed non-infective systemic inflammatory response syndrome (SIRS). MicroRNAs regulate cellular processes including inflammation and may be detected in blood. We aimed to establish definitive proof-of-principle that circulating microRNAs are differentially affected during sepsis and non-infective SIRS. Critically ill patients with severe (n = 21) or non-severe (n = 8) intra-abdominal sepsis; severe (n = 23) or non-severe (n = 21) non-infective SIRS; or no SIRS (n = 16) were studied. Next-generation sequencing and qRT-PCR were used to measure plasma microRNAs. Detectable blood miRNAs (n = 116) were generally up-regulated in SIRS compared to no-SIRS patients. Levels of these ‘circulating inflammation-related microRNAs’ (CIR-miRNAs) were 2.64 (IQR: 2.10–3.29) and 1.52 (IQR: 1.15–1.92) fold higher for non-infective SIRS and sepsis respectively (p < 0.0001), hence CIR-miRNAs appeared less abundant in sepsis than in SIRS. Six CIR-miRNAs (miR-30d-5p, miR-30a-5p, miR-192-5p, miR-26a-5p, miR-23a-5p, miR-191-5p) provided good-to-excellent discrimination of severe sepsis from severe SIRS (0.742–0.917 AUC of ROC curves). CIR-miRNA levels inversely correlated with pro-inflammatory cytokines (IL-1, IL-6 and others). Thus, among critically ill patients, sepsis and non-infective SIRS are associated with substantial, differential changes in CIR-miRNAs. CIR-miRNAs may be regulators of inflammation and warrant thorough evaluation as diagnostic and therapeutic targets.

Sepsis is defined as a systemic inflammatory response syndrome (SIRS) driven by infection^1^. Severe sepsis (sepsis accompanied by acute organ dysfunction) is a leading cause of death worldwide and the most common cause of death among patients on Intensive Care Units (ICUs)^2^. Critically ill patients may also develop SIRS driven by non-infective processes such as tissue ischemia. Sepsis and non-infective SIRS are hard to differentiate clinically but have very different therapeutic implications.

Understanding the immunopathology of sepsis and non-infective SIRS is vital for the development of novel diagnostics and treatments and for the appropriate use of antibiotics and novel immunotherapies. Much is known about the inflammatory responses which underlie sepsis and biomarkers of inflammation have been identified^3^. However there is growing recognition of the importance of anti-inflammatory, regulatory processes which accompany SIRS and sepsis^4^. These are physiological in that they terminate inflammation during recovery, and may be pathological causing sepsis-related immunosuppression. Ultimately, understanding how SIRS and sepsis are regulated may be key to developing novel biomarkers and interventions^5^.

MicroRNAs (miRNAs) are small (~22 nt) regulatory RNAs that function as post-transcriptional gene regulators^6^,^7^. Out of the 2588 described human miRNAs (miRBase.org), approximately 200 miRNAs have been reported circulating in the human blood in health and disease^8^,^9^,^10^. The impact of disease on circulating miRNAs has been assessed principally in the context of cancer^11^,^12^,^13^. A handful of studies have measured miRNAs present in the blood of sepsis patients, and reported conflicting findings^14^,^15^,^16^,^17^,^18^,^19^,^20^,^21^. Other studies have assessed specific candidate miRNAs based on leukocyte inflammatory responses in experimental models^22^,^23^,^24^,^25^,^26^. Advances in array and sequencing technologies now allow genome-wide screening for miRNAs, directly in human serum and plasma^27^,^28^, but studies which have taken this approach have been hampered by lack of robust normalization to properly quantify miRNA levels, small sample sizes and heterogeneous patient populations^14^,^17^.

In this study, we set out to establish definitive proof-of-principle that circulating microRNAs are differentially affected during systemic inflammation in critically ill patients depending on etiology. We studied a cohort of patients robustly characterized at the time of admission to critical care^29^ and selected tightly defined groups of patients with sepsis, non-infective SIRS, and critical illness without SIRS (no-SIRS patients). We used next-generation sequencing (NGS^30^) to identify normalizer miRNAs (present at consistent levels between patient groups) and to establish a long-list of candidate miRNAs differentially present in the blood of patients with sepsis, non-infective SIRS and without SIRS. We then used miRNA qRT-PCR to validate the most differentiating miRNAs and explore their performance in distinguishing sepsis from non-infective SIRS used singly and in combination.

We find that there is a general increase in the levels circulating miRNAs in the blood of patients with sepsis or non-infective SIRS compared with controls, and that this is more marked for non-infective SIRS than sepsis patients. We term these circulating inflammation-related miRNAs (CIR-miRNAs). Furthermore we find that the levels of CIR-miRNAs are differentially affected in sepsis and non-infective SIRS. Of note, among sepsis and SIRS patients, the blood levels of CIR-miRNAs inversely correlate with the plasma levels of key pro-inflammatory mediators such as interleukin-(IL)-1, IL-6, IL-8 and C-reactive protein (CRP), previously identified in systemic inflammation and sepsis^3^,^29^. CIR-miRNAs may be anti-inflammatory regulators of inflammation in patients with sepsis or non-infective SIRS and further investigation of their function and potential as disease markers and therapeutic targets is warranted.

## Materials and Methods

### Study Population

Patients comprised unselected adult admissions to a mixed medical/surgical intensive/high-dependency care unit (ICU/HDU) at an English acute hospital (Brighton and Sussex University Hospitals NHS Trust). For each patient, we gathered data describing demographics, reason for admission to ICU/HDU (i.e., medical/surgical patient), severity of illness (Sequential Organ Failure Assessment (SOFA) score in the first 24 hours), comorbidities, focus of infection, and routine clinical blood test results. Patients were categorized as having sepsis, non-infective SIRS or no-SIRS using standard criteria^29^. To minimize heterogeneity within groups, we included only patients with abdominal sepsis (defined as surgically or radiologically identified focus of infection within the abdomen) and defined distinct levels of severity: severe (SOFA ≥ 6) and non-severe (SOFA ≤ 3) sepsis/SIRS; patients with intermediate SOFA scores of 4–5 were excluded, defining 5 experimental groups (Table 1). Study blood samples were collected within a median time of 0.78 (IQR: 0.30–2.5; n = 89) hours of ICU admission with a maximum collection time of 5.8 hours. There was no statistical difference (p = 0.740, ANOVA) in time of sample collection between the groups (Table 1).

### Ethics Statement

Written informed consent or consultee approval to enroll was secured for all study participants. Under the UK Law (Mental Capacity Act) a consultee is someone (usually a next of kin, friend or family member) who gives approval for a patient who lacks capacity to be enrolled to a study, thus allowing such patients to participate in studies addressing the illness which affects them. This study was approved by the North Wales Research Ethics Committee (Central and East, reference 10/WNo03/19) and carried out in accordance with the approved guidelines. All data were anonymized.

### Preparation of NGS samples from plasma

Red blood cell (RBC) lysis can bias microRNA content in plasma^31^,^32^. The concentration of free hemoglobin ([Hb]) was measured in patient plasma by the Harboe spectrophotometric method^33^,^34^ and hemolytic^35^ samples with [Hb] > 0.6 g/L^36^ were excluded. Plasma pools were formed by combining equal volumes of patients’ plasma (Table 1). Total RNA was then extracted using the miRVana^TM^ PARIS^TM^ kit (Life Technologies)^13^. On average, we recovered 679 ± 165 pg RNA/μl plasma (mean ± SD); of this 849 ± 206 ng (mean ± SD) RNA (stored at −80 °C) were used for NGS technical duplicates. NGS cDNA libraries were prepared and validated at ARK Genomics (University of Edinburgh, UK) using specific barcodes for each cDNA library (Illumina TruSeq Small RNA sample) following manufacturer instructions. The libraries were finally eluted from gels, validated (for purity and correct insert size: 146 bp corresponding to ~22 nucleotides) on the High Sensitivity D1K ScreenTape (Agilent Technologies) and quantified by quantitative polymerase chain reaction (qPCR) prior to sequencing (Illumina HiSeq^TM^ 2500). In each lane, ~10^8^ NGS reads were acquired and, after filtering and sorting by library barcodes, sequences in any sample were mapped to miRBase (release 20) database. The resulting mapped reads (called counts) were arbitrarily normalized as miRNA counts/10^5^. See Supplementary Material and Methods for further detail. Raw sequencing data is available at the repository ArrayExpress (Accession number: E-MTAB-4637).

### Identification of miRNA normalizers in plasma

To identify miRNAs stably expressed in sepsis and SIRS relative to no-SIRS patients, for each average NGS miRNA count (technical duplicates, 5 groups representative of 89 individuals) we calculated: (i) percentage residual counts relative to average counts across groups and (ii) fold-differences (fd) between sepsis and SIRS (severe and non-severe). Normalizer candidates had: percentage residual counts within ± 20% when comparing any inflammatory disease group to no-SIRS and fd = 1.00 ± 0.20 (i.e. not more than 20% differential NGS counts in severe/non-severe sepsis vs SIRS).

### microRNA real-time qPCR array and cytokine analysis

Total plasma RNA was extracted from individual patients using the miRCURY™ RNA isolation-biofluids kit (Exiqon, Denmark). RNA was reverse transcribed (RT) and cDNA analyzed using the miRCURY LNA™ Universal RT microRNA PCR, Polyadenylation and cDNA synthesis method (Exiqon, see Supplementary Material and Methods for details). Each microRNA was assayed by qPCR (microRNA Ready-to-Use PCR, Pick-&-Mix using ExiLENT SYBR^®^ Green master mix) in 2 independent technical repeats including negative controls (no-template from the RT reaction) using a LightCycler^®^ 480 Real-Time PCR System (Roche). In each experimental group, ≥8 biological replicates were included. Assays returning 3 crossing point (Cp) values less than the negative control and Cp < 37 were accepted. The stability values of candidate normalizers were assessed using the ‘NormFinder’ software^37^. Any qPCR data was normalized to the average Cp of internal normalizers detected in all samples (delta Cp, dCp = normalizer Cp–assay Cp). Cytokines were measured on Luminex LX200 as before^29^ (see Supplementary Material and Methods).

### Statistical analyses

Datasets were analyzed using the GraphPad Prism 6 and/or IBM SPSS Statistics 22 software. The D’Agostino and Pearson omnibus and/or Shapiro-Wilk tests were used to test normal data distribution. If not normally distributed, medians with interquartile ranges (IQR, rather than means and standard deviation, SD) are shown and Mann-Whitney U Test (rather than t-tests) were used to calculate p-values in 2-group comparisons. Significances across more than 2 groups were assessed by ANOVA (Kruskal-Wallis test). For the qPCR miRNA array dataset, a Benjamini-Hochberg p-value correction^38^ was used to control for the number of false positives. The CIR-miRNA score (generated by binary logistic regression to predict SIRS vs sepsis) linearly combined the top performing 6 miRNA measurements in severe sepsis/SIRS patients. Multivariable analysis to correct for the effects of confounding variables was conducted using hierarchical multiple regression and hierarchical binary logistic regression (refer to Supplementary Materials and Methods for details). Correlations between CIR-miRNA scores and plasma levels of inflammatory mediators were evaluated using the Spearman rho and significances of the correlations. miRNA and cytokine analyses were conducted blind to the clinical data.

## Results

### Patients

Plasma samples used in the study came from 91 critically ill patients. Two samples with free hemoglobin (Hb) > 0.6 g/L were excluded as hemolytic from further analysis (Supplementary Fig. 1A), leaving 89 samples divided into five groups (Supplementary Fig. 1B): severe sepsis (n = 21); non-severe sepsis (n = 8); severe non-infective SIRS (n = 23); non-severe non-infective SIRS (n = 21); and patients without SIRS (no-SIRS controls, n = 16). Data describing demographics, clinical characteristics, sampling, severity of illness and key inflammatory biomarkers for study participants are shown in Table 1.

The median age of the patients was 66 years (IQR 54–75 years), 38 (43%) were male. Groups were well matched for age and gender (p = 0.229 and p = 0.638 respectively). By definition all 29 sepsis patients had an abdominal focus and all had surgical or radiological interventions. Non-sepsis patients were more heterogeneous with 39/60 (65%) being surgical (cancer surgery 17, abdominal surgery 16, cardiac surgery 4, and trauma 2), whilst the remainder were mixed medical patients. Patients with severe sepsis and severe non-infective SIRS had similar SOFA scores (Mean ± SD: 8.19 ± 2.68 and 7.56 ± 2.31 respectively, p = 1.00) which were markedly higher than in patients with non-severe disease 1.13 ± 0.99 and 1.29 ± 0.96 (p < 0.0001 both for sepsis and non-infective SIRS). Patients with severe sepsis had markedly higher levels of C-reactive protein (CRP: 161.8 ng/ml; IQR 109.5–215.7 ng/ml) and procalcitonin (PCT: 8.8 ng/ml; IQR 2.15–39.2 ng/ml) than patients with severe SIRS (CRP: 5.50 ng/ml; IQR 1.90–18.2 ng/ml, p < 0.0001 and PCT: 0.20 ng/ml; IQR 0.10–1.10 ng/ml, p = 0.0002). PCT levels also tended to be lower among patients with mild rather than severe sepsis (1.40 ng/ml; IQR 0.32–8.82 ng/ml) but this was not statistically significant (p = 1).

### Identification of plasma inflammation-related miRNAs by NGS

**Figure 1:**
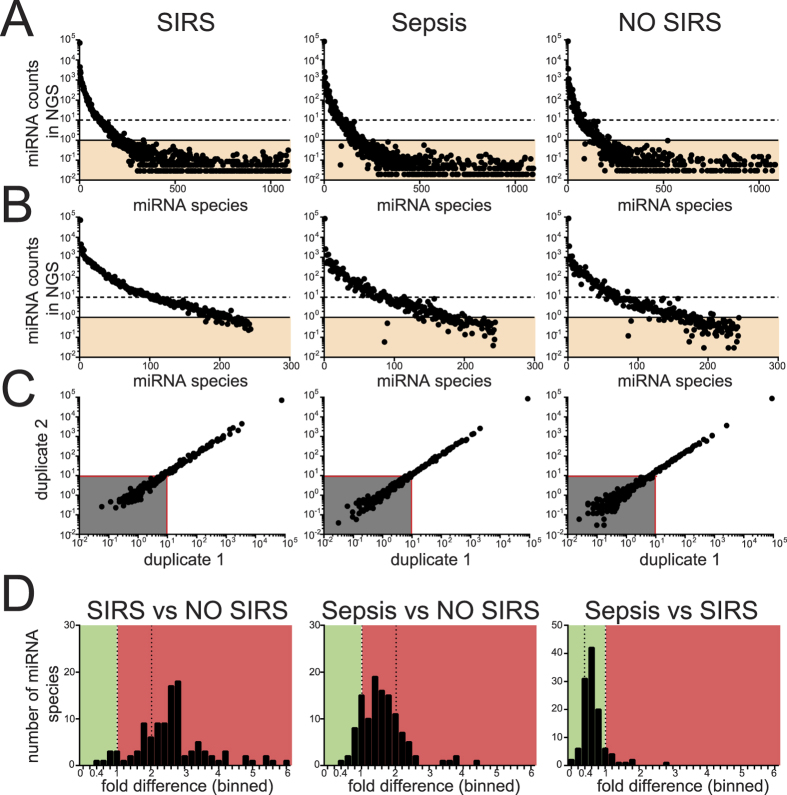
Patients plasma tested for miRNAs in Illumina NGS. Plasma total RNA was extracted from 10 pools (representative of 89 ICU patients, as in Table 1) using the miRVana PARIS technology and then human miRNAs were sequenced using the Illumina next generation sequencing (NGS) platform. (**A**) Representative plots show the number of blood miRNAs (x-axis, sorted based on their abundance in the first duplicate of SIRS) and relative NGS counts (y-axis), in SIRS, sepsis and no-SIRS patients. Many miRNA were expressed below 1/10^5^ NGS counts (orange shadowed areas) consistently across all pools and were excluded from further analysis. (**B**) Prolife of miRNA distribution after miRNAs with <1/10^5^ counts (orange areas) in all pools were excluded. (**C**) miRNA counts in 2 identical replicates are shown in scatter plots for SIRS, sepsis and no-SIRS patients. Reproducible results were obtained for miRNAs with NGS counts >10/10^5^ (red lines) and miRNA in the grey area were excluded. (**D**) The average miRNA counts (shortlisted in A–C, n = 116) from severe SIRS and Sepsis groups was expressed as a ratio against no-SIRS controls (left and middle panels) or in between each others (right panel), resulting in fold differences (fd) for each blood miRNA (histograms). Green and red areas, respectively, represent miRNA decrease and increase, separated by fd = 1 (left dotted line) and fd = +2 (right dotted line). Compared to no-SIRS, many CIR-miRNAs had fd > +2 in SIRS (left), but not in sepsis (fd < +2, middle). When Sepsis/SIRS are compared CIR-miRNAs are mostly downregulated (right).

In order to identify which of the currently known 2588 human miRNAs are found in the blood of critically ill patients with and without SIRS, we used NGS to sequence and differentially quantitate miRNAs in plasma pools comprising all patients in five groups (Table 1). Plasma pools were preferred to individual samples because they decrease the impact of individual outliers on the analysis. To control for the bias of miRNAs from RBC in differential analysis, we compiled pools so that average levels of hemolysis were comparable (Supplementary Fig. 1B and Table 1). Total RNA was then extracted from equal volumes of plasma and technical duplicates of cDNA libraries for Illumina NGS created. Results from 10 pools representative of 89 individuals are shown in Fig. 1. On average, NGS reads/library were 7.94 ± (SD) 1.36 × 10^6^ and miRBase-mapped reads (counts) were 43.5 ± (SD) 7.2%. Just below half of human microRNAs listed in miRBase.org (1097) returned counts in NGS (Fig. 1A) and were similarly distributed in each group. miRNAs present at low levels (<1 read per 10^5^ NGS counts across all five groups; Fig. 1A, orange area) showed high variability between the technical replicates and were excluded from further analysis. 244 miRNAs were expressed above >1/10^5^ counts (Fig. 1B) in at least one group and among these concordance between replicates was poor if average counts were <15/10^5^ (Fig. 1C, grey area) consistently across groups, leaving 116 miRNAs for subsequent analysis (Fig. 1D and Supplementary Table 1).

For each miRNA, fold differences (fd) were calculated comparing average counts in severe SIRS and sepsis with no-SIRS patients (Fig. 1D). Highly significant differences were seen when comparing plasma from patients with non-infective SIRS and patients with sepsis relative to no-SIRS controls, with the median fd for miRNAs being 2.64 (IQR: 2.10–3.29) and 1.52 (IQR: 1.15–1.92) respectively (p < 0.0001 and n = 116 for each comparison). We henceforth term the blood miRNAs that are affected in sepsis and non-infective SIRS, circulating inflammation-related miRNAs (CIR-miRNAs). In addition, when directly comparing sepsis and non-infective SIRS, CIR-miRNA levels were markedly lower in sepsis patients (median fd = 0.53; IQR: 0.45–0.74) (Fig. 1D, right panel).

### Identification of normalizer miRNAs

**Figure 2:**
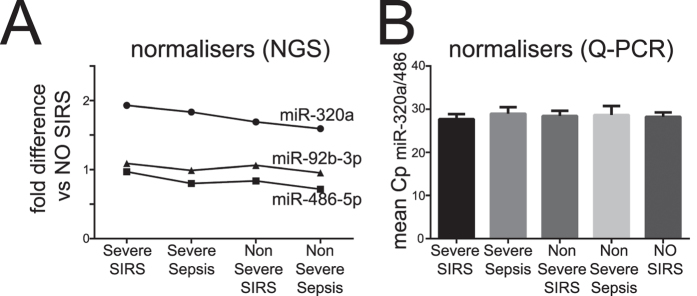
Shortlisting of internal normalizers in NGS and miRNA Q-PCR arrays. Plasma total RNA extracted and analyzed in NGS as described in Fig. 1, in 10 duplicate plasma pools (5 groups representative of 89 individuals) (**A**) or in 89 individuals samples using the miRCURY LNA Universal RT microRNA PCR technology (**B**). (**A**) Among the finally shortlisted miRNAs (miR-320a, miR-92-3p and miR-486-5p), the fold-differences (fd) of average NGS counts seen in severe and non-severe sepsis and SIRS groups (8 pools representative of 73 individuals) relative to no-SIRS controls (2 duplicate pools, n = 16) are shown. (**B**) In miRNA qPCR arrays, normalizer miRNAs were tested for 89 individual patients in 5 groups: severe sepsis (n = 21); non-severe sepsis (n = 8); severe SIRS (n = 23); non-severe SIRS (n = 21); and patients without SIRS (no-SIRS controls, n = 16), in two independent technical repeat experiments. Because miR-92b-3p was below the level of detection in 22/89 patients, it was excluded from further analysis. The mean Cp of the miR-320a and miR-486-5p is shown in each group and was selected as an internal normalizer for the miRNA qPCR array dataset.

To allow for robust comparison of miRNA levels in blood between individual samples we used NGS data to identify normalizer miRNAs present at consistent levels across the four inflammatory disease groups (Fig. 2A, n = 73) relative to the no-SIRS (n = 16) controls. This identified three candidate normalizers: miR-320a, miR-92b-3p and miR-486-5p that were expressed at stable levels across all inflammatory patient groups with levels varying by <20% (Fig. 2A).

We validated these candidate NGS normalizers using qPCR miRNA arrays on individual patient samples (n = 89, Fig. 2B). While miR-92b-3p was below the level of detection in 22/89 patients, the optimal normalizer (by NormFinder stability value^37^) was the average Cp of miR-320a and miR-486-5p. This performed better than any other single miRNA detected and was consistent across all 89 patients (Fig. 2B).

### Identification of a set of miRNAs which discriminate severe sepsis from SIRS

We applied qRT-PCR to validate the NGS results and determine if the general decrease in CIR-miRNAs observed in sepsis compared with non-infective SIRS could be used to distinguish these states at an individual patient level.

To maximize the possibility to detect reliable candidates we selected CIR-miRNAs for high level of detection in blood (by excluding CIR-miRNAs with consistently less than 35/10^5^ NGS counts in any group) and with fd ≤ 0.66 or fd ≥ 1.5 (when comparing sepsis to SIRS), leaving a panel of 47 CIR-miRNAs (including normalizers) to be validated in 89 patient samples. For each patient sample, Cp of single miRNAs were compared to the mean Cp of internal normalizers, to give delta-Cp (dCp). In parallel, we scored hemolysis in qPCR miRNA arrays as miR-23a/miR-451a ratio and excluded one sample that scored >7^39^ from further analysis (Supplementary Fig. 2).

**Figure 3:**
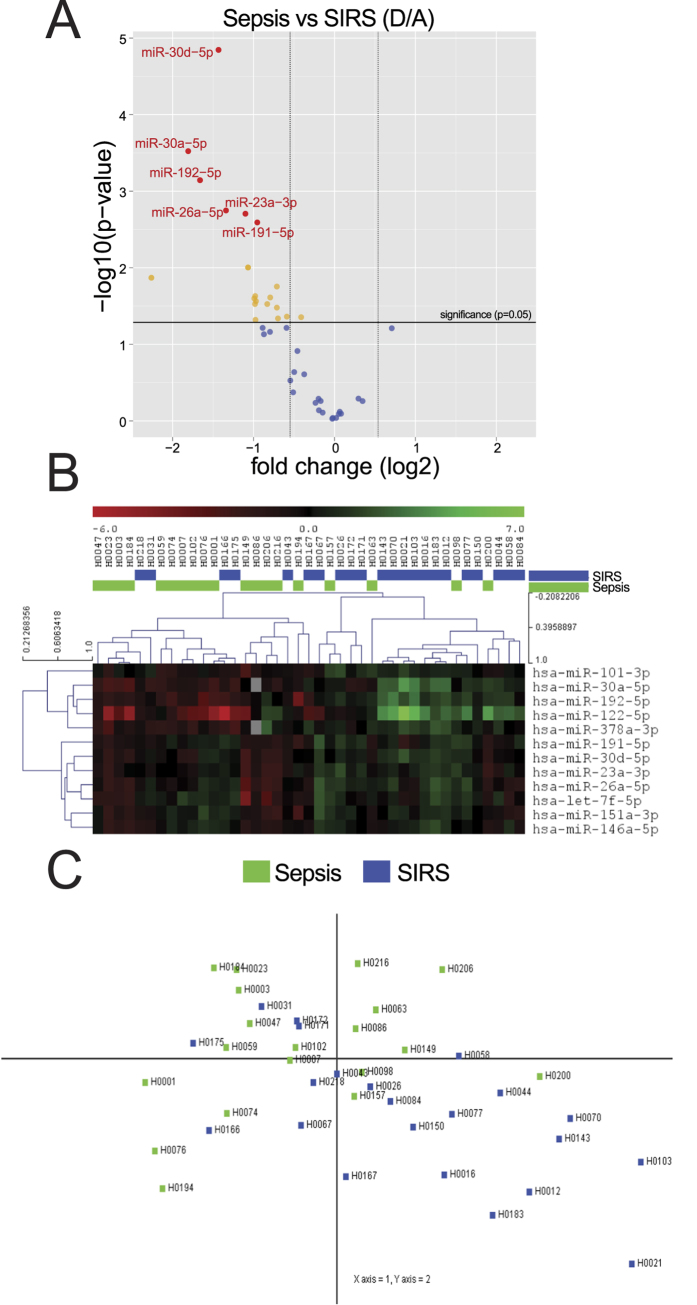
Shortlisted CIR-miRNAs measured with Exiqon miRNA qPCR arrays. In miRNA qPCR arrays, within each patient’s specimen, Cp of a single miRNA is compared to the mean Cp of 2 normalizers (as from Fig. 2) to give delta-Cp (dCp). dCp of all patients are analyzed, comparing severe Sepsis (D, n = 21) and SIRS (A, n = 23). (**A**) Volcano plot shows fold changes (log2, D/A) relative to p values (−log10) in each miRNA assay. In the upper left quadrant of the plot, around 20 miRNAs are significantly (red and yellow dots above the horizontal black line, which indicates a level of significance p ≤ 0.05) downregulated in D/A (fd < −1.5, left vertical line), see Table 2. Orange and red dots represent significant differences by t-test (p < 0.05) with red dots representing miRNA that also passed the Benjamini-Hochberg correction. No CIR-miRNA significantly increased in D/A. (**B**) Heatmap shows the top 12 significant miRNA clustering with opposite patterns in D/A. (**C**) Principal component analysis (PCA) transforms the top 5 significant miRNAs to maximize the visualization of differences across the severe sepsis and SIRS groups. The PCA plot shows that within the dataset it is possible to discriminate patients with SIRS (blue dots, mostly in the lower right quadrant of the PCA plot) away from patients with sepsis (Fig. 3C, green dots falling in other quadrants).

dCp were analyzed of patients with severe Sepsis (n = 21, group D in Fig. 3) and non-infective SIRS (n = 23, group A in Fig. 3). Confirming our NGS analysis (Fig. 3 and Table 2), the majority (~94%) of CIR-miRNAs had negative fold changes in qPCR analysis in the sepsis group compared with non-infective SIRS (Volcano plot in Fig. 3A). Table 2 lists 20 CIR-miRNAs that were statistically significantly decreased (t-test, p < 0.05) including the top 6 differentially expressed that passed the Benjamini-Hochberg correction^38^ (respectively, the yellow and red dots above the horizontal black line, p ≤ 0.05, and with fd ≤ −1.5, left vertical line in Fig. 3A). No CIR-miRNAs were statistically significantly increased in severe sepsis compared to severe non-infective SIRS (Fig. 3A). Furthermore, the top-12 significantly different CIR-miRNAs showed inverse patterns in sepsis and SIRS (heatmap in Fig. 3B).

To determine whether severe sepsis and non-infective SIRS are associated with distinct patterns of change in CIR-miRNAs we conducted a principal component analysis. A combination of top 5 significantly different CIR-miRNAs (miR-30d-5p, miR-30a-5p, miR-192-5p, miR-26a-5p and miR-23a-5p) was sufficient to achieve discrimination of severe sepsis from non-infective SIRS patients (Fig. 3C).

**Figure 4:**
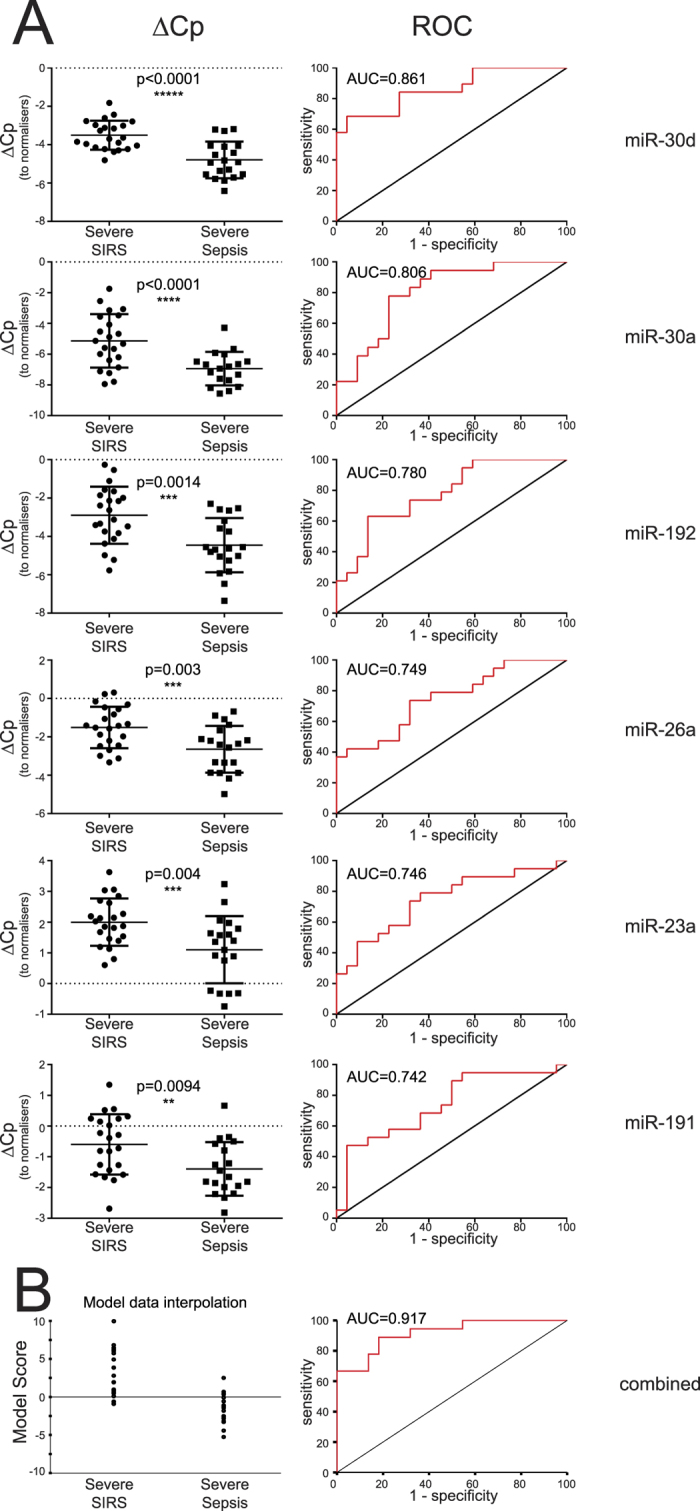
CIR-miRNAs are good-to-excellent biomarkers of sepsis. In miRNA qPCR arrays, data was analyzed as in Fig. 3 and the top-6 differentially expressed miRNA in sepsis compared to SIRS (after the Benjamini-Hochberg correction) are shown. (**A**) Left dot plots show dCp values in severe SIRS vs sepsis in individual samples (n = 21 and n = 23 for sepsis and SIRS respectively, except n = 20 in sepsis for miRNA-30a-5p) together with the level of significance. The relative receiver operator curve (ROC, right) is shown with the Area Under the Curve (AUC). Each of the top 6 significant CIR-miRNAs is a good-to-excellent biomarker and CIR-miRNAs were mostly downregulated in Sepsis compared to SIRS in Exiqon miRNA qPCR arrays. (**B**) A model combines the top-6 significant CIR-miRNAs to maximize distinction between SIRS and sepsis. The CIR-miRNA score is directly related to the odds of having SIRS or sepsis given the measurements of the 6 top miRNAs (see Material and Methods for further details). Left dot plot shows the model interpolation of the experimental cohort: SIRS patients -that have high CIR-miRNA levels (in A)- tend to score >0, whilst sepsis patients tend to score <0. ROC (and AUC, right) shows that the 6 CIR-miRNAs combined outperformed single miRNAs.

Relative to normalizers, dCp values in severe SIRS were significantly higher than in sepsis (Fig. 4A), indicating that CIR-miRNAs are more abundant in SIRS than in sepsis patients. When the data were plotted as receiver operator curves (ROC) each of the top 6 significantly different CIR-miRNAs (additionally including miRNA-191-5p) provided good to excellent discrimination with areas under the curve (AUC) between 0.742 to 0.861 (Fig. 4A). Hierarchical logistic regression (Supplementary Fig. 4A) confirmed that the predictive value of these 6 miRNAs is retained after controlling for SOFA score; age; sex; patient survival outcome and time of sample collection.

We further created a model combining levels of these 6 CIR-miRNAs into a score that maximized the distinction between non-infective SIRS and sepsis (Fig. 4B). In the model interpolation of the cohort, non-infective SIRS and sepsis patients tended to score respectively >0 and <0; hence the higher the model score the more likely patients are to have non-infective SIRS rather than sepsis, as described by a concomitant increase of multiple CIR-miRNAs (CIR-miRNA score). The ROC curves with AUC 0.917 (Fig. 4B, right) and AUC 0.89 (after multivariable correction analysis, as shown in Supplementary Fig. 4) for the model interpolation data show that the top-6 significant CIR-miRNAs combined together outperformed any single miRNA. Moreover, CIR-miRNAs either combined or used singularly mimicked and even outperformed the traditional sepsis biomarker, PCT (Supplementary Fig. 3).

### Levels of CIR-miRNAs are inversely correlated with that of inflammatory cytokines

**Figure 5:**
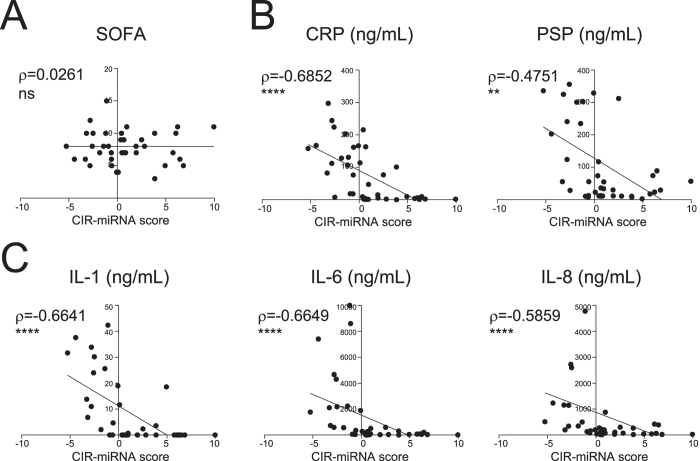
Correlation of the model scores with pathology scores and plasma levels of immune mediators relevant in sepsis and SIRS. The model scores that combine the top-6 CIR-miRNA measurements in Severe SIRS and Severe Sepsis patients were plotted against (**A**) the pathology score (SOFA, sequential organ failure assessment); (**B**) current biomarkers of sepsis and inflammation, CRP (C-reactive protein) and PSP (pancreatic soluble protein); and (**C**) inflammatory cytokines, IL-6, IL-8, and IL-1. Correlation trends are shown with the linear regression model including Spearman rho and the significances of the correlations. Negative scores -typical of sepsis patients with lower plasma CIR-miRNAs (as in Fig. 4)- correspond to individuals with increased levels of inflammatory mediators. Positive scores -more often seen in SIRS patients and reflective of high plasma CIR-miRNAs- are found in individuals with low levels of inflammatory cytokines. Thus, CIR-miRNA levels negatively correlate with pro-inflammatory cytokines critical in systemic inflammatory conditions.

We obtained CIR-miRNA scores as a mathematical function of the plasma levels of 6 CIR-miRNAs found to be consistently reduced in sepsis (and preferentially leading to score <0). The CIR-miRNA scores were then correlated to plasma levels of pro-inflammatory mediators, and SOFA severity scores, across sepsis and SIRS patients (Fig. 5). CIR-miRNA scores did not correlate with SOFA scores (Fig. 5A). However, CIR-miRNA scores negatively correlated with levels of pro-inflammatory mediators, suggesting that a marked increase of multiple CIR-miRNAs is significantly associated with low levels of pro-inflammatory markers (CRP and PSP, Fig. 5B) and cytokines (IL-1, IL-8 and IL-6, Fig. 5C). Thus, in severe inflammatory disease, the levels of CIR-miRNAs change in the opposite direction to levels of pro-inflammatory mediators.

## Discussion

We have undertaken a robust exploratory evaluation of circulating miRNAs among critically ill patients with sepsis and non-infective SIRS in comparison with internal control patients. We identified circulating inflammation-related miRNAs (CIR-miRNAs) that are broadly increased in both sepsis and non-infective SIRS patients when compared with no-SIRS controls. Among patients with severe sepsis, CIR-miRNA levels were lower than in patients with comparably severe *non-infective* SIRS. Furthermore, we have identified six CIR-miRNAs which were markedly reduced in sepsis compared with non-infective SIRS and performed well as discriminatory markers of these conditions. Combined together, these CIR-miRNAs were more discriminatory than traditional sepsis biomarkers such as PCT (Supplementary Fig. 3) and CRP^29^. Notably, we found that CIR-miRNA levels correlate inversely with pro-inflammatory mediators.

Previous studies exploring the effect of sepsis and SIRS on circulating miRNAs have typically studied individual miRNAs shortlisted from mouse models or human cells^21^, stimulated with lipopolysaccharide (LPS)^22^,^23^,^24^,^25^,^26^, made comparisons with healthy controls^14^,^18^,^19^ (rather than SIRS patients^15^,^16^,^19^,^21^) or comparisons within sepsis patients for prognostic implications^17^,^18^,^20^. Often, these studies had small patient cohorts (analyzed as individuals) and did not account for the nature of the underlying infection during sepsis and for severity of illness^14^,^15^,^16^,^17^,^18^,^19^,^20^,^21^, both affecting the inflammatory response^40^. Only in two previous studies^14^,^17^, have circulating miRNAs been sought primarily with a genome-wide approach (with miRNA microarrays^14^ being less sensitive than NGS). Many previous studies^14^,^15^,^16^,^17^,^18^,^19^,^20^,^21^ have suffered from lack of rigorous normalization^41^ and bias coming from hemolysis (promoting the release miRNAs from RBCs^31^,^32^).

Our study overcomes such limitations providing a definitive evaluation of CIR-miRNAs in human sepsis and SIRS. We propose a novel approach to the normalization of blood miRNAs. First, we excluded the bias of RBC-derived miRNAs form our analysis by balancing free Hb levels across the experimental groups and -unlike in any previous study^14^,^17^,^41^- we normalized CIR-miRNAs against endogenous normalizing plasma miRNAs^42^ consistently expressed across all recruited individuals. The best normalizer for our dataset was a combination of miR-320a and miR-486-5p (while miR-92b-3p was excluded because its levels fell below the detection limit of qPCR in many individuals). Interestingly, miR-320a and miR-486-5p were included in the 5 best miRNAs for least concentration variations in plasma and/or serum in a recent detailed study of blood miRNAs (with n = 12 individuals) by an independent group^9^, suggesting that these miRNAs may be useful endogenous normalizers. Additionally, miR-486-5p is one of the most abundant miRNAs in RBC^9^ and its blood amounts may derive mostly by RBC^31^. Hence, it is not surprising that, after balancing haemolysis across the samples prior to miRNA analysis, we found relative stability of miR-486-5p in the individuals from our cohort. This supports that a two-stage normalization (Hb and endogenous miRNA) approach may enable the use of RBC-derived miRNAs as endogenous normalizers in future studies.

Interestingly none of the previously proposed “biomarkers of sepsis” was included in our best CIR-miRNAs that discriminate sepsis from SIRS, except for miR-23a (also found in^14^). We however confirmed modulation of miR-146a, miR-122, miR-223, and the Let-7 family- which collectively showed a tendency to decrease in sepsis compared to SIRS (Table 2). Hence, in contrast to previous reports we did not find increased levels of miR-223 and miR-146a^14^,^17^,^18^,^43^,^44^ associated with sepsis over SIRS. These differences likely result from the fact that previous studies compared sepsis patients directly to healthy individuals (rather than SIRS patients). In agreement, relative to control patients (no-SIRS), we also found that CIR-miRNAs are generally up-regulated in sepsis, thus reconciling our study with previous literature. Further our study is compatible with a previous report^16^ in which both miR-223 and miR-146a are reduced in sepsis compared to SIRS. Interestingly, 6/7 miRNAs investigated in the same study also showed a tendency to decrease in sepsis compared to SIRS. Other miRNAs previously proposed as “biomarkers of sepsis”, including miR-15a and miR-16^19^, miR-150^14^,^21^ (in agreement with^15^), and miR-4772-5p-iso^21^ were not confirmed in our independent cohort.

As we used the CRP to define our experimental groups, we cannot provide a direct comparison of the biomarker performance of CIR-miRNAs and CRP in this study. Nevertheless, within our own data the combination of the top 6 CIR-miRNAs outperformed the most discriminatory existing marker, PCT (Supplementary Fig. 3)^29^ and exceeds previously published performance of CRP^29^. The time of sample collection, sex, survival outcome, age and SOFA scores did not significantly affect the measurements of shortlisted CIR-miRNAs (Supplementary Fig. 4). Importantly, the predictive value of the top 6 CIR-miRNAs (either used alone or in combination) was generally well-preserved after controlling for a number of confounding variables in our cohort (Supplementary Fig. 4). However, our study observations remain limited to a single cohort of patients and as such constitute a “hypothesis generating” study. A larger prospective study will be needed to consolidate the proposed CIR-miRNAs as disease biomarkers in a different population. For instance, it should be noted that the small number of recruited patients with non-severe sepsis (n = 8) precluded the statistical analysis of the non-severe groups and future research will be needed to assess how CIR-miRNA levels are affected by disease severity. After matching for age, severity of the disease, time of sample collection, sex and free Hb, our study populations showed an intrinsic heterogeneity in the surgical to medical patient ratio (as 100% abdominal sepsis patients underwent surgery, decreased to average 65% in the control groups). Future studies should assess the impact of the focus of infection and surgery on levels of CIR-miRNAs.

It is presently unclear whether any of the top 6 CIR-miRNAs play a role in sepsis and SIRS. However, miR-191 and miR-26a have been recently associated with inflammatory and autoimmune conditions in humans and mice^45^,^46^,^47^. Particularly, miR-26a was reported to limit inflammatory responses possibly by promoting regulatory T cell responses^46^ or through NF-kB inhibition in chondrocytes^47^. Also miR-23a has been implicated in inflammation via multiple pathways and it may: attenuate the cytotoxic activity in CD8 T cells^48^; limit excessive T cell immunopathology by regulating reactive oxygen species^49^; and/or regulate pro-inflammatory cytokine expression following TLR-signal in macrophages, via the NF-kB pathway^50^. Kinases and transcription factors important in immune-cell differentiation and regulation such as Blimp-1^51^, p53/MDM2^52^ and PTEN^53^ may be targets of miR-30a, miR-30d, miR-192 and miR26a, all CIR-miRNAs downregulated in sepsis, in our study. Further research in our laboratory is underway to evaluate whether and how these genes are relevant in sepsis.

**Figure 6:**
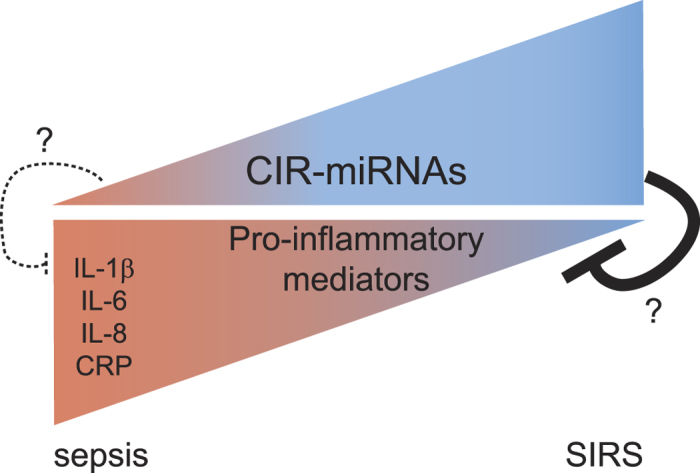
Proposed model for CIR-miRNA and inflammatory mediator plasma levels. The triangular shapes represent plasma levels of CIR-miRNA (circulating miRNA, top) and pro-inflammatory mediators (bottom). Based on our results, in Sepsis we found low levels of CIR-miRNAs correlating with increasing levels of pro-inflammatory mediators (dark red). In contrast, in SIRS patients CIR-miRNAs are more abundant than what is found for sepsis patients correlating with lower levels of pro-inflammatory markers (blue). We speculate that immunologically relevant CIR-miRNAs may exist that act as regulators of inflammatory processes especially during systemic inflammatory diseases. This hypothesis is consistent with recent data showing that regulatory cells secrete exosomes which exert miRNA-mediated immune-suppression.

We provide evidence that will support future studies defining the origin of CIR-miRNAs, whether they traffic in blood inside exosomes (vesicles) or in complexes with Argonaute^8^,^54^, their targets and function. The magnitude of the changes we have observed in inflammation and the correlations with inflammatory cytokines suggest that CIR-miRNAs originate in cells of the immune system^55^,^56^. We find that levels of CIR-miRNAs inversely correlate with levels of inflammatory cytokines that are typically elevated in sepsis such as IL-1β, IL-6, and IL-8, and CRP^3^,^57^. This opens up the possibility that CIR-miRNAs may be part of the anti-inflammatory response^4^ suppressing immune cell activation in severe sepsis and inflammation (Fig. 6). This hypothesis is compatible with the recent discovery that (murine) regulatory T cells suppress inflammatory responses by secreting a number of miRNAs analogous to the human CIR-miRNAs found in this study^56^. How exactly the CIR-miRNAs fit into the inflammatory cascade in sepsis and SIRS remains to be determined in future studies. For example, miRNAs may be detected by Toll-like receptors as danger signals^58^,^59^ and transferred to antigen presenting cells^60^, NK^59^ and T^56^ cells to drive cell-differentiation. Also, individual cellular miRNAs were shown to regulate inflammatory signaling cascades leading to immune cell activation or suppression (reviewed in^61^,^62^,^63^ and^50^).

In summary, among critically ill patients, elevation of CIR-miRNAs is a hallmark of systemic inflammation. Quantitative and qualitative differences in CIR-miRNA levels exist in sepsis and non-infective SIRS. Our study provides proof-of-principle that sepsis and non-infective SIRS are associated with distinct changes in CIR-miRNAs which may be exploited for novel therapeutic and diagnostic approaches, whilst future investigations of the role of CIR-miRNAs in the immunopathogenesis of sepsis and non-infective SIRS are warranted.

## Additional Information

**How to cite this article**: Caserta, S. *et al*. Circulating Plasma microRNAs can differentiate Human Sepsis and Systemic Inflammatory Response Syndrome (SIRS). *Sci. Rep.* **6**, 28006; doi: 10.1038/srep28006 (2016).

## Supplementary Material

Supplementary Information

## Figures and Tables

**Table 1:** Demographics, severity of illness and key inflammatory biomarkers of the patients used in the study.

**Group Name**	**n**	**Age (years)**	SOFAscores	Surgical:Medical ratio	Time to sample(hours)	**CRP (ng/ml)**	**PCT (ng/ml)**	**F:M ratio (♀%)**	[Hb] mg/Lplasma
Severe SIRS	23	58.5 ± 17.0	7.56 ± 2.31	6:17	0.73(IQR: 0.22–2.3)	19.8 ± 32.7	1.73 ± 3.85	12:11 (52.2%)	155 ± 110
Severe Sepsis	21	67.7 ± 12.9	8.19 ± 2.68	21:0	0.88(IQR: 0.33–2.5)	156 ± 76.9	21.9 ± 28.7	13:8 (61.9%)	215 ± 145
Non-severe SIRS	21	61.0 ± 14.0	1.29 ± 0.96	18:3	0.67(IQR: 0.41–2.8)	20.6 ± 34.3	0.29 ± 0.34	12:9 (57.1%)	216 ± 159
Non-severe Sepsis	8	58.1 ± 23.3	1.13 ± 0.99	8:0	1.6(IQR: 0.40–4.2)	159 ± 69.5	5.77 ± 9.66	3:5 (37.5%)	115 ± 96.3
NO SIRS	16	64.9 ± 14.6	4.19 ± 3.41	15:1	0.70(IQR: 0.14–2.4)	19.2 ± 20.3	0.77 ± 1.00	11:5 (68.7%)	160 ± 133

**Table 2:** Significant differentially-expressed miRNAs in severe Sepsis (D) compared to SIRS (A).

**miRNA**	SDdCq D	SDdCq A	MeandCq D	MeandCq A	FdD/A	**p-value**	BH adj.p-value
hsa-miR-30d-5p	1.08	0.76	−4.94	−3.51	−2.70	0.000014	0.00061
hsa-miR-30a-5p	1.09	1.74	−6.94	−5.13	−3.50	0.00030	0.0064
hsa-miR-192-5p	1.45	1.49	−4.56	−2.90	−3.16	0.00072	0.010
hsa-miR-26a-5p	1.49	1.08	−2.85	−1.51	−2.53	0.0018	0.017
hsa-miR-23a-3p	1.29	0.77	0.90	2.00	−2.14	0.0020	0.017
hsa-miR-191-5p	0.96	0.98	−1.55	−0.60	−1.94	0.0026	0.018
hsa-let-7f-5p	1.52	0.96	−4.23	−3.16	−2.10	0.0099	0.061
hsa-miR-122-5p	2.49	3.22	−2.85	−0.59	−4.80	0.014	0.073
hsa-miR-101-3p	1.05	0.81	−2.92	−2.20	−1.64	0.018	0.084
hsa-miR-30c-5p	1.57	1.09	−4.07	−3.09	−1.97	0.023	0.085
hsa-miR-151a-3p	1.28	0.82	−4.16	−3.36	−1.73	0.024	0.085
hsa-miR-378a-3p	1.44	1.25	−4.16	−3.17	−1.99	0.025	0.085
hsa-miR-223-3p	1.50	1.26	3.30	4.27	−1.96	0.027	0.085
hsa-miR-27b-3p	1.76	0.92	−2.93	−1.95	−1.98	0.030	0.085
hsa-let-7a-5p	1.47	0.81	−0.69	0.14	−1.78	0.030	0.085
hsa-miR-146a-5p	1.22	0.84	−1.71	−1.00	−1.64	0.033	0.089
hsa-miR-107	0.97	0.87	−2.45	−1.86	−1.50	0.043	0.10
hsa-let-7b-5p	0.67	0.63	−0.96	−0.54	−1.33	0.044	0.10
hsa-miR-30e-3p	1.05	1.12	−6.65	−5.96	−1.62	0.046	0.10
hsa-miR-143-3p	1.73	1.30	−4.54	−3.57	−1.97	0.048	0.10

## References

[b1] American College of Chest Physicians/Society of Critical Care Medicine Consensus Conference: definitions for sepsis and organ failure and guidelines for the use of innovative therapies in sepsis. *Crit. Care Med.* **20**, 864–874 (1992). 1597042

[b2] Angus D. C. . Epidemiology of severe sepsis in the United States: analysis of incidence, outcome, and associated costs of care. Crit. Care Med. 29, 1303–1310, doi: 10.1097/00003246-200107000-00002 (2001). 11445675

[b3] Adib-Conquy M. & Cavaillon J. M. Stress molecules in sepsis and systemic inflammatory response syndrome. FEBS Lett. 581, 3723–3733, doi: 10.1016/j.febslet.2007.03.074 (2007). 17428476

[b4] Oberholzer A., Oberholzer C. & Moldawer L. L. Sepsis syndromes: understanding the role of innate and acquired immunity. Shock 16, 83–96, doi: 10.1097/00024382-200116020-00001 (2001). 11508871

[b5] Lichtenstern C., Brenner T., Bardenheuer H. J. & Weigand M. A. Predictors of survival in sepsis: what is the best inflammatory marker to measure? Curr. Opin. Infect. Dis. 25, 328–336, doi: 10.1097/QCO.0b013e3283522038 (2012). 22421751

[b6] Bartel D. P. MicroRNAs: genomics, biogenesis, mechanism, and function. Cell 116, 281–297, doi: 10.1016/S0092-8674(04)00045-5 (2004). 14744438

[b7] Bartel D. P. MicroRNAs: target recognition and regulatory functions. Cell 136, 215–233, doi: 10.1016/j.cell.2009.01.002 (2009). 19167326 PMC3794896

[b8] Arroyo J. D. . Argonaute2 complexes carry a population of circulating microRNAs independent of vesicles in human plasma. Proc. Natl. Acad. Sci. USA 108, 5003–5008, doi: 10.1073/pnas.1019055108 (2011). 21383194 PMC3064324

[b9] Wang K. . Comparing the MicroRNA spectrum between serum and plasma. PLoS One 7, e41561, doi: 10.1371/journal.pone.0041561 (2012). 22859996 PMC3409228

[b10] Duttagupta R., Jiang R., Gollub J., Getts R. C. & Jones K. W. Impact of cellular miRNAs on circulating miRNA biomarker signatures. PLoS One 6, e20769, doi: 10.1371/journal.pone.0020769 (2011). 21698099 PMC3117799

[b11] Mitchell P. S. . Circulating microRNAs as stable blood-based markers for cancer detection. Proc. Natl. Acad. Sci. USA 105, 10513–10518, doi: 10.1073/pnas.0804549105 (2008). 18663219 PMC2492472

[b12] Reid G., Kirschner M. B. & van Zandwijk N. Circulating microRNAs: Association with disease and potential use as biomarkers. Crit. Rev. Oncol. Hematol. 80, 193–208, doi: 10.1016/j.critrevonc.2010.11.004 (2011). 21145252

[b13] Jones C. I. . Identification of circulating microRNAs as diagnostic biomarkers for use in multiple myeloma. Br. J. Cancer 107, 1987–1996, doi: 10.1038/bjc.2012.525 (2012). 23169280 PMC3516695

[b14] Vasilescu C. . MicroRNA fingerprints identify miR-150 as a plasma prognostic marker in patients with sepsis. PLoS One 4, e7405, doi: 10.1371/journal.pone.0007405 (2009). 19823581 PMC2756627

[b15] Roderburg C. . Circulating microRNA-150 serum levels predict survival in patients with critical illness and sepsis. PLoS One 8, e54612, doi: 10.1371/journal.pone.0054612 (2013). 23372743 PMC3555785

[b16] Wang J. F. . Serum miR-146a and miR-223 as potential new biomarkers for sepsis. Biochem. Biophys. Res. Commun. 394, 184–188, doi: 10.1016/j.bbrc.2010.02.145 (2010). 20188071

[b17] Wang H. . Serum microRNA signatures identified by Solexa sequencing predict sepsis patients’ mortality: a prospective observational study. PLoS One 7, e38885, doi: 10.1371/journal.pone.0038885 (2012). 22719975 PMC3376145

[b18] Wang H. J. . Four serum microRNAs identified as diagnostic biomarkers of sepsis. J Trauma Acute Care Surg 73, 850–854, doi: 10.1097/TA.0b013e31825a7560 (2012). 23026916

[b19] Wang H. . Evidence for serum miR-15a and miR-16 levels as biomarkers that distinguish sepsis from systemic inflammatory response syndrome in human subjects. Clin. Chem. Lab. Med. 50, 1423–1428, doi: 10.1515/cclm-2011-0826 (2012). 22868808

[b20] Wang H. . Serum miR-574-5p: a prognostic predictor of sepsis patients. Shock 37, 263–267, doi: 10.1097/SHK.0b013e318241baf8 (2012). 22344312

[b21] Ma Y. . Genome-wide sequencing of cellular microRNAs identifies a combinatorial expression signature diagnostic of sepsis. PLoS One 8, e75918, doi: 10.1371/journal.pone.0075918 (2013). 24146790 PMC3797812

[b22] Tacke F. . Levels of circulating miR-133a are elevated in sepsis and predict mortality in critically ill patients. Crit. Care Med. 42, 1096–1104, doi: 10.1097/CCM.0000000000000131 (2014). 24413579

[b23] Li Y. . Plasticity of leukocytic exudates in resolving acute inflammation is regulated by MicroRNA and proresolving mediators. Immunity 39, 885–898, doi: 10.1016/j.immuni.2013.10.011 (2013). 24238341 PMC3888517

[b24] Wu S. C. . Profiling circulating microRNA expression in experimental sepsis using cecal ligation and puncture. PLoS One 8, e77936, doi: 10.1371/journal.pone.0077936 (2013). 24205035 PMC3813489

[b25] Hsieh C. H. . Whole blood-derived microRNA signatures in mice exposed to lipopolysaccharides. J. Biomed. Sci. 19, 69, doi: 10.1186/1423-0127-19-69 (2012). 22849760 PMC3419134

[b26] Sun X. . MicroRNA-181b regulates NF-kappaB-mediated vascular inflammation. J. Clin. Invest. 122, 1973–1990, doi: 10.1172/JCI61495 (2012). 22622040 PMC3366408

[b27] Jensen S. G. . Evaluation of two commercial global miRNA expression profiling platforms for detection of less abundant miRNAs. BMC Genomics 12, 435, doi: 10.1186/1471-2164-12-435 (2011). 21867561 PMC3184117

[b28] Kang K., Peng X., Luo J. & Gou D. Identification of circulating miRNA biomarkers based on global quantitative real-time PCR profiling. J Anim Sci Biotechnol 3, 4, doi: 10.1186/2049-1891-3-4 (2012). 22958414 PMC3415128

[b29] Llewelyn M. J. . Sepsis biomarkers in unselected patients on admission to intensive or high-dependency care. Crit. Care 17, R60, doi: 10.1186/cc12588 (2013). 23531337 PMC3672658

[b30] Metzker M. L. Sequencing technologies-the next generation. Nat Rev Genet 11, 31–46, doi: 10.1038/nrg2626 (2010). 19997069

[b31] Pritchard C. C. . Blood cell origin of circulating microRNAs: a cautionary note for cancer biomarker studies. Cancer Prev. Res. (*Phila*.) 5, 492–497, doi: 10.1158/1940-6207.CAPR-11-0370 (2012). PMC4186243 22158052

[b32] Kirschner M. B. . Haemolysis during sample preparation alters microRNA content of plasma. PLoS One 6, e24145, doi: 10.1371/journal.pone.0024145 (2011). 21909417 PMC3164711

[b33] Harboe M. A method for determination of hemoglobin in plasma by near-ultraviolet spectrophotometry. Scand. J. Clin. Lab. Invest. 11, 66–70, doi: 10.3109/00365515909060410 (1959). 13646603

[b34] Adamzik M. . Free hemoglobin concentration in severe sepsis: methods of measurement and prediction of outcome. Crit. Care 16, R125, doi: 10.1186/cc11425 (2012). 22800762 PMC3580706

[b35] Han V., Serrano K. & Devine D. V. A comparative study of common techniques used to measure haemolysis in stored red cell concentrates. Vox Sang. 98, 116–123, doi: 10.1111/j.1423-0410.2009.01249.x (2010). 19719459

[b36] Lippi G., Salvagno G. L., Montagnana M., Brocco G. & Guidi G. C. Influence of hemolysis on routine clinical chemistry testing. Clin. Chem. Lab. Med. 44, 311–316, doi: 10.1515/CCLM.2006.054 (2006). 16519604

[b37] Andersen C. L., Jensen J. L. & Orntoft T. F. Normalization of real-time quantitative reverse transcription-PCR data: a model-based variance estimation approach to identify genes suited for normalization, applied to bladder and colon cancer data sets. Cancer Res. 64, 5245–5250, doi: 10.1158/0008-5472.CAN-04-0496 (2004). 15289330

[b38] Benjamini Y. & Hochberg Y. Controlling the False Discovery Rate: A Practical and Powerful Approach to Multiple Testing. J R Stat Soc Series B Stat Methodol 57, 289–300, doi: 10.2307/2346101 (1995).

[b39] Blondal T. . Assessing sample and miRNA profile quality in serum and plasma or other biofluids. Methods 59, S1–6, doi: 10.1016/j.ymeth.2012.09.015 (2013). 23036329

[b40] Mayr F. B. . Infection rate and acute organ dysfunction risk as explanations for racial differences in severe sepsis. JAMA 303, 2495–2503, doi: 10.1001/jama.2010.851 (2010). 20571016 PMC3910506

[b41] Benz F. . U6 is unsuitable for normalization of serum miRNA levels in patients with sepsis or liver fibrosis. Exp. Mol. Med. 45, e42, doi: 10.1038/emm.2013.81 (2013). 24052167 PMC3789266

[b42] Marabita F. . Normalization of circulating microRNA expression data obtained by quantitative real-time RT-PCR. Brief Bioinform 17, 204–212, doi: 10.1093/bib/bbv056 (2016). 26238539 PMC4793896

[b43] Nahid M. A., Pauley K. M., Satoh M. & Chan E. K. miR-146a is critical for endotoxin-induced tolerance: IMPLICATION IN INNATE IMMUNITY. J. Biol. Chem. 284, 34590–34599, doi: 10.1074/jbc.M109.056317 (2009). 19840932 PMC2787321

[b44] Taganov K. D., Boldin M. P., Chang K. J. & Baltimore D. NF-kappaB-dependent induction of microRNA miR-146, an inhibitor targeted to signaling proteins of innate immune responses. Proc. Natl. Acad. Sci. USA 103, 12481–12486, doi: 10.1073/pnas.0605298103 (2006). 16885212 PMC1567904

[b45] Nagpal N. & Kulshreshtha R. miR-191: an emerging player in disease biology. Frontiers in genetics 5, 99, doi: 10.3389/fgene.2014.00099 (2014). 24795757 PMC4005961

[b46] Ma H., Zhang S., Shi D., Mao Y. & Cui J. MicroRNA-26a Promotes Regulatory T cells and Suppresses Autoimmune Diabetes in Mice. Inflammation 39, 1–9, doi: 10.1007/s10753-015-0215-0 (2016). 26208605

[b47] Xie Q. . Reciprocal inhibition between miR-26a and NF-kappaB regulates obesity-related chronic inflammation in chondrocytes. Biosci. Rep. 35, e00204, doi: 10.1042/BSR20150071 (2015). 26182366 PMC4613702

[b48] Chandran P. A. . The TGF-beta-inducible miR-23a cluster attenuates IFN-gamma levels and antigen-specific cytotoxicity in human CD8(+) T cells. J. Leukoc. Biol. 96, 633–645, doi: 10.1189/jlb.3A0114-025R (2014). 25030422

[b49] Zhang B. . MicroRNA-23a Curbs Necrosis during Early T Cell Activation by Enforcing Intracellular Reactive Oxygen Species Equilibrium. Immunity 44, 568–581, doi: 10.1016/j.immuni.2016.01.007 (2016). 26921109 PMC4794397

[b50] Peng P., Li Z. & Liu X. Reduced Expression of miR-23a Suppresses A20 in TLR-stimulated Macrophages. Inflammation 38, 1787–1793, doi: 10.1007/s10753-015-0156-7 (2015). 25832477

[b51] Wang X. . PRDM1 is directly targeted by miR-30a-5p and modulates the Wnt/beta-catenin pathway in a Dkk1-dependent manner during glioma growth. Cancer Lett. 331, 211–219, doi: 10.1016/j.canlet.2013.01.005 (2013). 23348703

[b52] Pichiorri F. . Downregulation of p53-inducible microRNAs 192, 194, and 215 impairs the p53/MDM2 autoregulatory loop in multiple myeloma development. Cancer Cell 18, 367–381, doi: 10.1016/j.ccr.2010.09.005 (2010). 20951946 PMC3561766

[b53] Huse J. T. . The PTEN-regulating microRNA miR-26a is amplified in high-grade glioma and facilitates gliomagenesis *in vivo*. Genes Dev. 23, 1327–1337, doi: 10.1101/gad.1777409 (2009). 19487573 PMC2701585

[b54] Hunter M. P. . Detection of microRNA expression in human peripheral blood microvesicles. PLoS One 3, e3694, doi: 10.1371/journal.pone.0003694 (2008). 19002258 PMC2577891

[b55] O’Connell R. M., Rao D. S., Chaudhuri A. A. & Baltimore D. Physiological and pathological roles for microRNAs in the immune system. Nat. Rev. Immunol. 10, 111–122, doi: 10.1038/nri2708 (2010). 20098459

[b56] Okoye I. S. . MicroRNA-containing T-regulatory-cell-derived exosomes suppress pathogenic T helper 1 cells. Immunity 41, 89–103, doi: 10.1016/j.immuni.2014.05.019 (2014). 25035954 PMC4104030

[b57] Lever A. & Mackenzie I. Sepsis: definition, epidemiology, and diagnosis. BMJ 335, 879–883, doi: 10.1136/bmj.39346.495880.AE (2007). 17962288 PMC2043413

[b58] Fabbri M. TLRs as miRNA receptors. Cancer Res. 72, 6333–6337, doi: 10.1158/0008-5472.CAN-12-3229 (2012). 23222301

[b59] He S. . MicroRNAs activate natural killer cells through Toll-like receptor signaling. Blood 121, 4663–4671, doi: 10.1182/blood-2012-07-441360 (2013). 23580661 PMC3674667

[b60] Mittelbrunn M. . Unidirectional transfer of microRNA-loaded exosomes from T cells to antigen-presenting cells. Nat Commun 2, 282, doi: 10.1038/ncomms1285 (2011). 21505438 PMC3104548

[b61] O’Connell R. M., Rao D. S. & Baltimore D. microRNA regulation of inflammatory responses. Annu. Rev. Immunol. 30, 295–312, doi: 10.1146/annurev-immunol-020711-075013 (2012). 22224773

[b62] Ha T. Y. The Role of MicroRNAs in Regulatory T Cells and in the Immune Response. Immune Netw. 11, 11–41, doi: 10.4110/in.2011.11.1.11 (2011). 21494372 PMC3072673

[b63] Tang X. . MicroRNA networks in regulatory T cells. J. Physiol. Biochem. 70, 869–875, doi: 10.1007/s13105-014-0348-x (2014). 25108555

