# Supplementary material for: Circulating Plasma microRNAs can differentiate Human Sepsis and Systemic Inflammatory Response Syndrome (SIRS)
Source: Sci Rep. 2016 Jun 20;6:28006. doi: 10.1038/srep28006 (PMC4913253; doi:10.1038/srep28006)
Supplement: Supplementary Information [file srep28006-s1.pdf]

Circulating Plasma microRNAs can differentiate Human Sepsis and Systemic Inflammatory Response Syndrome (SIRS).

Stefano Caserta*[1], Florian Kern[1], Jonathan Cohen[1], Stephen Drage[2], Sarah F. Newbury[1], Martin J. Llewelyn[1,2]

[1]Brighton and Sussex Medical School, Falmer, East Sussex, United Kingdom, BN1 9PS;

[2]Brighton and Sussex University Hospitals NHS Trust, Eastern Road, Brighton, United Kingdom BN2 5BE

***Correspondence to:**

Stefano Caserta. Division of Clinical Medicine, BSMS - Brighton and Sussex Medical School, Medical Research Building, University of Sussex, Falmer BN1 9PS, UK

Phone: +44-(0)1273-877886; Fax: +44-(0)1273 877889; E-mail: s.caserta@bsms.ac.uk

Supplementary Material Contents:

1. **Supplementary Figure 1 with Legend** **pages 2-3**
2. **Supplementary Figure 2 with Legend** **pages 4-5**
3. **Supplementary Figure 3 with Legend** **pages 6-7**
4. **Supplementary Figure 4 with Legend** **pages 8-10**
5. **Supplementary Table 1** **pages 11-13**
6. **Supplementary Materials and Methods** **pages 14-19**
7. **Supplementary References** **pages 20-21**

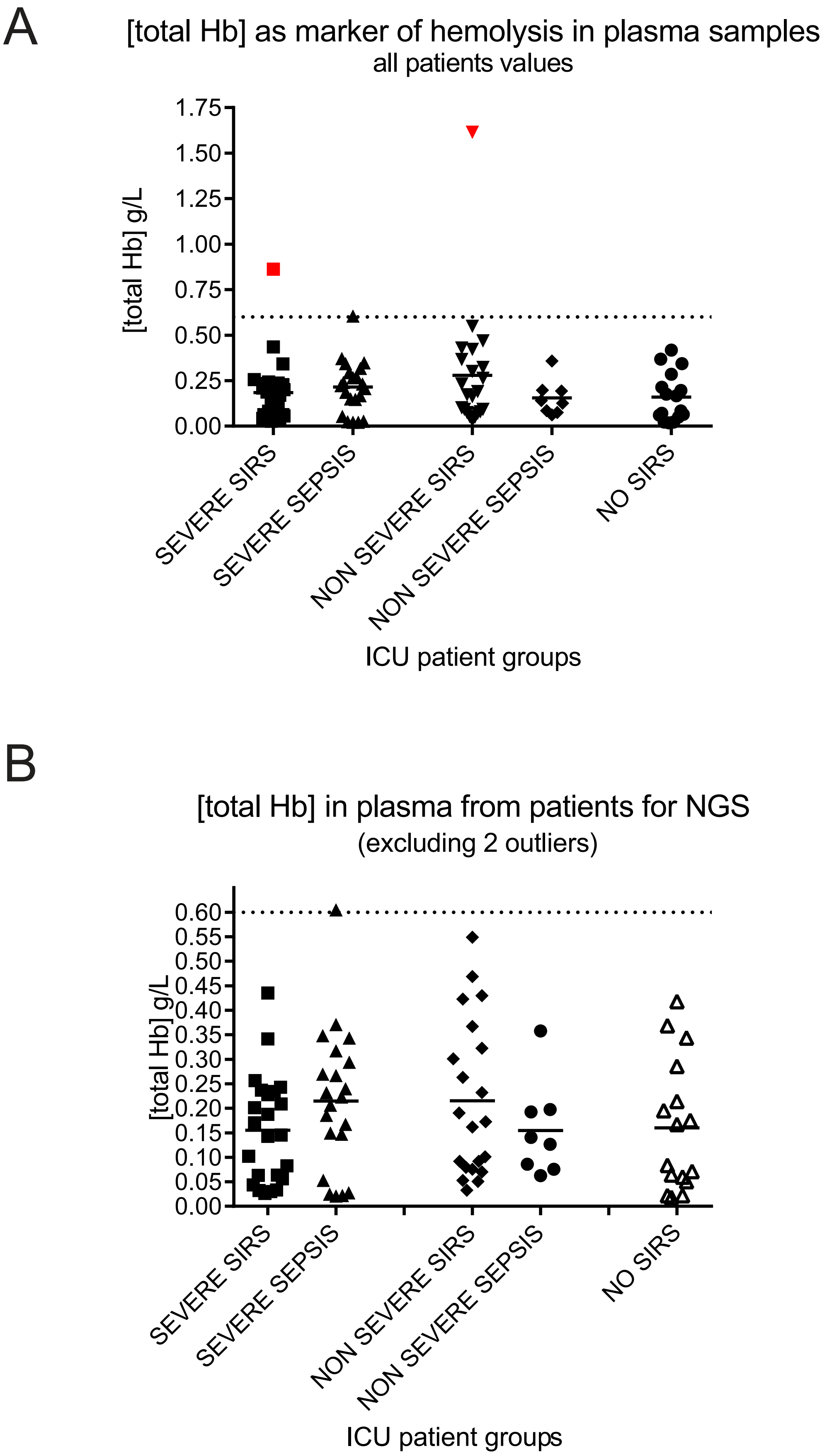

Supplementary Figure 1

1 Supplementary Figure 1 with Legend

Supplementary Figure 1. Exclusion of hemolytic samples and average hemoglobin levels in the experimental cohort. A. Hemolysis, which is marked in plasma if free hemoglobin (Hb) levels >0.6g/L, was measured by Harboe spectrophotometric method in any sample (n=91). Shown measurements are the average of 3 technical replicates/patient. Hemolytic samples (red; 1 in the severe SIRS groups and 1 in the non-severe SIRS) were excluded from the study. **B.** Hb levels are shown in any experimental group used in NGS and miRNA Q-PCR array, after the exclusion of outliers. Importantly, average Hb did not differ significantly across groups, suggesting that RBC lysis is equally represented across the experimental groups prior to the NGS analysis. RBCs may be responsible for miRNA presence in the blood. Balancing the levels of miRNAs across experimental groups may also positively affect normalization. In fact in our analysis, the internal normalizer miR-486-5p is one of the most abundant miRNA circulating in blood and is mostly derived from RBC.

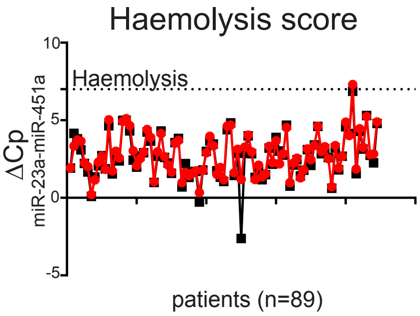

Supplementary Figure 2

2 Supplementary Figure 2 with Legend

Supplementary Figure 2. Independent validation of hemolysis levels in miRNA qPCR arrays. In Exiqon miRNA qPCR arrays, hemolysis is scored as a ratio of the Cp of miR-23a/miR-451a assays in two independent, technical repeat experiments - shown respectively in the black and in the red lines for each individual sample (x-axis, n=89). If the miR-23a/miR-451a ratio cut off >7 is reached samples are excluded from any further analysis. In agreement with our previous spectrophotometric analysis (Supplementary Figure 1), the qPCR platform confirmed similar levels of hemolysis across the groups and only 1 patient sample in the severe sepsis group was deemed to be excluded from further analysis.

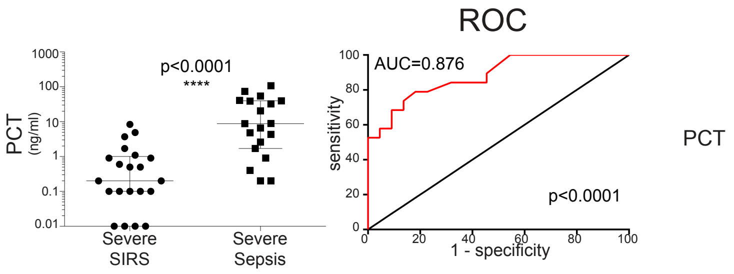

Supplementary Figure 3

3 Supplementary Figure 3 with Legend

Supplementary Figure 3. Performance of traditional biomarker PCT in the Severe SIRS and Sepsis cohorts. Left dot plots show PCT levels (ng/ml) in severe SIRS vs sepsis in individual samples (n=21 and n=23 for sepsis and SIRS respectively) together with the level of significance. The relative receiver operator curve (ROC, right) is shown with the Area Under the Curve (AUC).

A

ROC

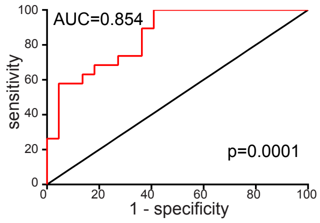

Hierarchical logistic regression levels	Variables considered	R square (% variance of independent variable)	p (level of significance)
1st	SOFA; Sex; Age; Survival outcome; Time of sample collection	0.122 (12.2%)	0.375 (ns)
2nd	miR-30d	0.424 (42.4%)	0.001 (***)

miR-30d

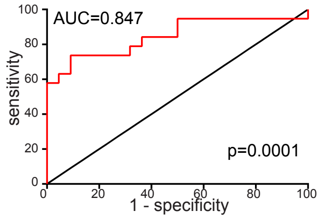

Hierarchical logistic regression levels	Variables considered	R square (% variance of independent variable)	p (level of significance)
1st	SOFA; Sex; Age; Survival outcome; Time of sample collection	0.129 (12.9%)	0.356 (ns)
2nd	miR-30a	0.418 (41.8%)	0.001 (***)

miR-30a

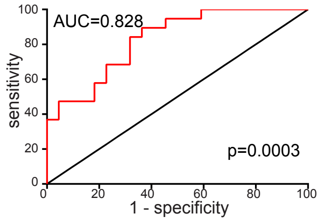

Hierarchical logistic regression levels	Variables considered	R square (% variance of independent variable)	p (level of significance)
1st	SOFA; Sex; Age; Survival outcome; Time of sample collection	0.122 (12.2%)	0.375 (ns)
2nd	miR-192	0.339 (33.9%)	0.009 (**)

miR-192

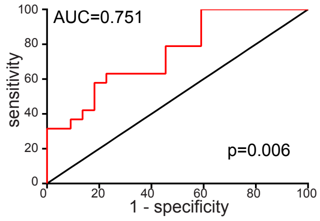

Hierarchical logistic regression levels	Variables considered	R square (% variance of independent variable)	p (level of significance)
1st	SOFA; Sex; Age; Survival outcome; Time of sample collection	0.122 (12.2%)	0.375 (ns)
2nd	miR-26a	0.293 (29.3%)	0.028 (*)

miR-26a

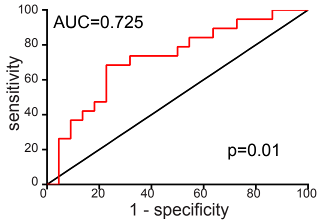

Hierarchical logistic regression levels	Variables considered	R square (% variance of independent variable)	p (level of significance)
1st	SOFA; Sex; Age; Survival outcome; Time of sample collection	0.122 (12.2%)	0.375 (ns)
2nd	miR-23a	0.293 (29.3%)	0.027 (*)

miR-23a

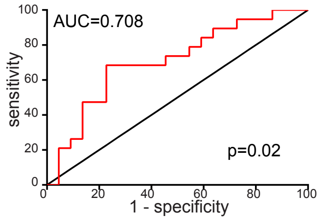

Hierarchical logistic regression levels	Variables considered	R square (% variance of independent variable)	p (level of significance)
1st	SOFA; Sex; Age; Survival outcome; Time of sample collection	0.122 (12.2%)	0.375 (ns)
2nd	miR-191	0.259 (25.9%)	0.056 (ns)

miR-191

B

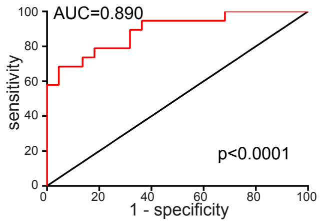

Hierarchical logistic regression levels	Variables considered	R square (% variance of independent variable)	p (level of significance)
1st	SOFA; Sex; Age; Survival outcome; Time of sample collection	0.129 (12.9%)	0.356 (ns)
2nd	miR-30d; miR-30a; miR-192; miR-26a; miR-23a; miR-191	0.535 (53.5%)	0.001 (***)

combined

Supplementary Figure 4

4 Supplementary Figure 4 with Legend

Supplementary Figure 4. Single or combined CIR-miRNAs discriminate Severe SIRS and Sepsis after correction for multiple confounding variables. Hierarchical binary logistic regression models of the Sepsis/SIRS predictive value were generated based on each of the top 6 CIR-miRNAs (**A**), and their combination (**B**), after controlling for a number of confounding variables. The confounding variables (SOFA score, age, sex, patient survival outcome, and time of sample collection) were introduced at the first level (block 1) of the regression models. Thereafter, in the 2nd level (block 2) of the analyses, each individual miRNAs (**A**) or the combination of the top 6 CIR-miRNAs (**B**) were assessed for the capacity to predict a significant amount of the variance of the probability of having Sepsis versus SIRS. In **A.** and **B.**, left panels show the receiver operator curves (ROC) of each model interpolation of the experimental cohorts (based on the relative regression equations), the level of significance of the difference between the interpolated cohorts, and the resulting Area Under the Curve (AUC). For each model, the tables (right) list the variables introduced at each step of the hierarchical binary logistic regression together with the resulting (Cox and Snell) R square values (the percentages of the variance in the predictive value) with levels of significance after the first and the second regression steps (i.e., the total model significance). R squares values show that, in any case, the confounding variables introduced at step 1 did not account significantly for the prediction of sepsis vs SIRS, as expected ($p>0.05$, 1st step). At the second step, all miRNAs significantly improved the prediction of sepsis and SIRS and could account for 26-42% (miRNAs used alone, **A**) or 53.5% (top 6 CIR-miRNAs combined, **B**) of the variance in the probability of having Sepsis/SIRS. This suggests that the predictive values of the top 6 CIR-

miRNA is preserved after controlling for a number of confounding variables. In the case of miR-191, even if the total significance of the model is p=0.056, the second step significantly improved the model prediction (p=0.008). It should be noted that since in our study SOFA and age were rigorously matched the interval of these variables is consequently limited (see Table 1). As patients were coming from the same ICU type, this was not deemed to be a confounding variable in our study. (n=21 and n=23 for sepsis and SIRS respectively, except n=20 in sepsis for miR-30a and the combined score)

5 Supplementary Table 1. The levels of 116 circulating miRNAs shortlisted after NGS in sepsis and SIRS.

miRNA ID	s.sepsis vs NO SIRS fd	s.SIRS vs NO SIRS fd	S.Sepsis vs S.SIRS fd	Notes*
hsa-miR-208b-3p	0.85	128.93	0.01	>15; <35
hsa-miR-133a-3p	0.63	20.47	0.03	>15; <35
hsa-miR-122-5p	1.09	6.66	0.16	
hsa-miR-10b-5p	0.49	2.40	0.21	
hsa-miR-3591-3p	1.53	7.35	0.21	
hsa-miR-378a-3p	2.27	9.13	0.25	
hsa-miR-192-5p	1.73	6.84	0.25	
hsa-miR-375	2.03	7.28	0.28	
hsa-miR-125b-2-3p	1.93	6.30	0.31	>15; <35
hsa-miR-30a-5p	0.73	2.36	0.31	
hsa-miR-10a-5p	1.09	3.35	0.32	
hsa-miR-181c-5p	2.11	5.91	0.36	>15; <35
hsa-miR-423-5p	1.16	3.18	0.36	
hsa-let-7f-5p	0.98	2.65	0.37	
hsa-miR-3184-3p	1.15	3.10	0.37	
hsa-miR-1307-5p	1.97	5.04	0.39	>15; <35
hsa-miR-26b-5p	1.03	2.63	0.39	
hsa-miR-142-3p	1.34	3.36	0.40	>15; <35
hsa-miR-150-5p	1.42	3.46	0.41	>15; <35
hsa-miR-30e-3p	1.14	2.78	0.41	>15; <35
hsa-let-7d-5p	0.99	2.40	0.41	>15; <35
hsa-miR-148a-5p	2.33	5.46	0.43	>15; <35
hsa-miR-27b-3p	1.82	4.24	0.43	
hsa-let-7a-5p	0.85	1.94	0.44	
hsa-miR-744-5p	1.17	2.66	0.44	
hsa-let-7c-5p	0.99	2.23	0.45	>15; <35
hsa-miR-28-3p	1.26	2.81	0.45	
hsa-miR-29a-3p	1.23	2.75	0.45	>15; <35
hsa-miR-1246	1.36	3.01	0.45	
hsa-miR-340-5p	1.31	2.89	0.45	
hsa-miR-181a-5p	1.92	4.11	0.47	
hsa-miR-222-3p	1.75	3.71	0.47	>15; <35
hsa-miR-181b-5p	1.51	3.20	0.47	
hsa-miR-98-5p	0.90	1.89	0.47	>15; <35
hsa-miR-409-3p	1.83	3.84	0.48	
hsa-miR-182-5p	0.87	1.82	0.48	
hsa-miR-423-3p	2.01	4.18	0.48	

hsa-miR-3184-5p	2.32	4.76	0.49	>15; <35
hsa-miR-148a-3p	1.81	3.70	0.49	
hsa-miR-144-5p	1.43	2.80	0.51	>15; <35
hsa-miR-769-5p	2.44	4.74	0.52	>15; <35
hsa-miR-342-3p	1.08	2.10	0.52	>15; <35
hsa-miR-410-3p	1.51	2.89	0.52	>15; <35
hsa-miR-26a-5p	1.35	2.54	0.53	
hsa-miR-22-3p	2.12	3.99	0.53	
hsa-miR-99a-5p	1.30	2.44	0.53	>15; <35
hsa-miR-103b	1.52	2.85	0.53	
hsa-miR-103a-3p	1.53	2.85	0.54	
hsa-miR-100-5p	1.20	2.23	0.54	>15; <35
hsa-miR-148b-3p	1.15	2.14	0.54	
hsa-miR-151a-3p	0.95	1.76	0.54	
hsa-miR-199b-3p	1.49	2.73	0.54	>15; <35
hsa-miR-27a-3p	1.99	3.64	0.55	
hsa-miR-151a-5p	1.40	2.55	0.55	
hsa-miR-126-3p	1.31	2.35	0.56	>15; <35
hsa-miR-199a-3p	1.66	2.86	0.58	>15; <35
hsa-miR-24-3p	1.13	1.93	0.58	>15; <35
hsa-miR-101-3p	1.63	2.78	0.58	
hsa-miR-30d-5p	1.48	2.51	0.59	
hsa-miR-191-5p	0.75	1.27	0.59	
hsa-let-7i-5p	1.56	2.63	0.59	
hsa-miR-143-3p	1.68	2.81	0.60	
hsa-miR-107	1.68	2.79	0.60	
hsa-miR-1307-3p	1.65	2.73	0.60	>15; <35
hsa-miR-150-3p	0.64	1.05	0.61	>15; <35
hsa-miR-451a	1.53	2.51	0.61	
hsa-let-7b-5p	1.31	2.11	0.62	
hsa-miR-127-3p	1.49	2.36	0.63	
hsa-miR-125a-5p	1.52	2.39	0.63	>15; <35
hsa-miR-130a-3p	1.41	2.23	0.63	
hsa-let-7d-3p	1.83	2.88	0.64	>15; <35
hsa-miR-140-3p	2.17	3.39	0.64	
hsa-miR-345-5p	3.54	5.53	0.64	>15; <35
hsa-miR-584-5p	1.07	1.67	0.64	
hsa-miR-21-5p	1.67	2.52	0.66	
hsa-miR-532-5p	2.21	3.32	0.66	>15; <35
hsa-miR-19b-3p	1.71	2.51	0.68	>15; <35
hsa-miR-146a-5p	1.30	1.88	0.69	
hsa-miR-93-5p	2.02	2.91	0.69	
hsa-miR-301a-3p	1.87	2.69	0.69	>15; <35
hsa-miR-128-3p	1.49	2.14	0.70	>15; <35

hsa-miR-223-3p	1.91	2.72	0.70	
hsa-miR-210-3p	3.83	5.42	0.71	>15; <35
hsa-miR-126-5p	0.76	1.08	0.71	
hsa-miR-25-3p	1.88	2.64	0.71	
hsa-miR-92a-3p	1.35	1.84	0.73	
hsa-miR-106b-3p	2.66	3.61	0.74	>15; <35
hsa-miR-146b-5p	1.38	1.85	0.74	
hsa-miR-186-5p	1.60	2.14	0.75	
hsa-miR-3074-5p	1.07	1.42	0.75	>15; <35
hsa-miR-23a-3p	1.72	2.24	0.77	>15; <35
hsa-miR-30e-5p	1.60	2.08	0.77	
hsa-miR-421	1.80	2.33	0.77	>15; <35
hsa-let-7g-5p	2.02	2.60	0.77	
hsa-miR-484	1.78	2.27	0.78	>15; <35
hsa-miR-142-5p	1.97	2.52	0.78	
hsa-miR-221-3p	1.49	1.84	0.81	
hsa-miR-320b	2.30	2.65	0.87	>15; <35
hsa-miR-425-5p	2.35	2.68	0.88	
hsa-miR-486-3p	1.28	1.45	0.88	
hsa-miR-21-3p	2.46	2.75	0.89	
hsa-miR-15a-5p	1.76	1.90	0.92	
hsa-miR-130b-3p	1.78	1.87	0.95	
hsa-miR-144-3p	0.80	0.79	1.02	>15; <35
**hsa-miR-92b-3p**	1.09	1.06	1.03	>15; <35
hsa-miR-363-3p	1.74	1.62	1.07	
hsa-miR-500a-3p	2.64	2.44	1.08	>15; <35
**hsa-miR-320a**	1.93	1.69	1.14	
hsa-miR-99b-5p	0.96	0.83	1.15	
**hsa-miR-486-5p**	0.97	0.84	1.16	
hsa-miR-16-5p	2.14	1.72	1.25	
hsa-miR-652-3p	3.72	2.53	1.47	>15; <35
hsa-miR-223-5p	4.49	2.76	1.62	>15; <35
hsa-miR-941	3.46	2.00	1.73	
hsa-miR-183-5p	0.92	0.53	1.74	>15; <35
hsa-miR-501-3p	1.00	0.36	2.78	>15; <35

*If miRNA NGS counts were more than $15/10^5$ but less than $35/10^5$ (light grey boxes) we did not generally proceed to the following Q-PCR validation. Candidate normalizers are shown in bold. miRNAs decreased or increased in sepsis compared to SIRS are highlighted respectively in green and in red boxes, whilst light blue boxes indicate potential normalizers.

6 Supplementary Materials and Methods

Sample handling and normalization of hemolysis

Study blood samples were collected in Na-citrate tubes from patients within 6 hours of ICU admission and centrifuged. Plasma was stored at -80°C until the day of analysis, thawed on ice and kept at 4°C until the RNA extraction. Plasma was stored at -80°C until the day of analysis, thawed on ice and kept at 4°C until the RNA extraction. Red blood cell (RBC) lysis during sample handling has the potential to bias microRNA content in plasma[1-4]. The concentration of free hemoglobin ([Hb]) in plasma reflects the degree of any hemolysis[5]. Free [Hb] in patient samples was assessed by the Harboe spectrophotometric method[6,7] and samples with [Hb]>0.6g/L were excluded from further analysis[8]. Briefly, the total [Hb] in a freshly prepared Hb standard was validated using SysMex SLS-technology[9] to detect any Hb form in the human blood. Standard dilutions and plasma samples (1:10) were tested in triplicate to determine the A415, A380, and A450 and the Harboe [oxy-Hb][5] was: [oxy-Hb] (g/l)=167x(A415) – 84x(A380) – 84x(A450). Harboe oxy-Hb and total Hb content of the standards were linearly interpolated to quantitate total Hb in each sample. In qPCR miRNA arrays, further assessment of hemolysis in individual samples was made by calculating the ratio of miR-23a to miR-451a and using a cut-off >7 to indicate significant hemolysis[10].

Plasma RNA extraction and NGS of plasma miRNA

After exclusion of hemolysis specimens, plasma pools were formed by combining equal volumes of patients' plasma in the groups of Table 1. Total RNA was extracted from 2.5 ml plasma using the miRVana^{TM} PARIS^{TM} technology kit (Life Technologies)[11]. Briefly, each sample was denatured and processed according to manufacturer's instructions to extract RNA with Acid-Phenol:$CHCl_3$; the recovered aqueous phase was mixed with ethanol (molecular biology grade; SIGMA; 1:1.25) and loaded onto replicate columns to bind RNA. After multiple column washes, RNA was eluted in 95°C DEPC-treated H_20 (Life Technologies) from replicate columns, pooled and quantified using a Nanodrop spectrophotometer. Typically we recovered 679±165 pg RNA/µl of plasma (mean±SD). On the same day, an average RNA input of 849±206 ng (mean±SD) was created for technical duplicates of NGS and stored at -80°C. Before cDNA library preparation for NGS, RNA preparations were validated for the presence of miRNA using a Taqman miRNA assay (Life Technologies) for human miR-16. NGS cDNA libraries were prepared and validated from plasma RNA by ARK Genomics (University of Edinburgh, UK), following manufacturer instructions, with specific barcodes for each cDNA library (Illumina TruSeq Small RNA sample protocol). Briefly, samples were ligated with an adapter (3' end) and a primer (5' end) before being reversely transcribed. The cDNA obtained was used as a template for PCR to add sample specific barcodes and extend adapters. Thereafter, samples were purified by electrophoresis (6% polyacrylamide gels) and bands corresponding to ~22 nucleotides in the original sample were size-selected (correct insert size: 146bp) after band staining and visualization under UV-light. The amplified size selected DNA was extracted from the gel by overnight soaking (H_20) and concentrated. The final preparation was checked for size and potential adapter-

dimer contamination by electrophoresis. The libraries were finally eluted from gels and run on the High Sensitivity D1K ScreenTape (Agilent Technologies) to determine size and purity prior to final quantification by qPCR and sequenced on a HiSeq™ 2500 Illumina instrument by loading duplicate libraries on separate lanes. In each lane, ~10^8 NGS reads were acquired and, after filtering and sorting by library barcodes, sequences in any sample were mapped to the miRBase (release 20) database. The resulting mapped reads (called counts) were arbitrarily normalized as miRNA counts/10^5.

qPCR miRNA array sample preparation

Total RNA was extracted from plasma of individual patients using the miRCURY™ RNA isolation - biofluids kit (Exiqon, Vedbaek, Denmark). Plasma was thawed on ice and centrifuged (3000g, 5 min, 4°C). For each sample, plasma (200 µL) was mixed with 60 µl of Lysis solution BF containing 1 µg carrier-RNA per 60µl Lysis Solution BF and RNA spike-in template mixture (UniSp4, UniSp3 and UniSp6). Samples were vortexed briefly and incubated 3 min at room temperature, before adding 20 µL Protein Precipitation solution BF. Samples were vortexed, incubated 1 min at room temperature and centrifuged (11000g, 3 min). Clear supernatants were mixed with isopropanol (270 µL, SIGMA), briefly vortexed and loaded onto binding columns. After multiple washes, RNA was eluted in RNase-free H_2O by centrifugation (11000g) and stored at -80°C.

microRNA real-time qPCR array and analysis

RNA (2 µl) was reverse transcribed using the miRCURY LNA™ Universal RT microRNA PCR, Polyadenylation and cDNA synthesis kit (Exiqon). cDNA (1:50) was

assayed in qPCR as by the miRCURY LNA™ Universal RT microRNA PCR protocol. Each microRNA was assayed once by qPCR (on the microRNA Ready-to-Use PCR, Pick-&-Mix using ExiLENT SYBR® Green master mix) in 2 independent technical repeat experiments including negative controls (no-template from the reverse transcription reaction). In each experimental group, ≥8 biological replicates were included. The amplification was performed in a LightCycler® 480 Real-Time PCR System (Roche) in 384-well plates. The amplification curves were analyzed using the Roche LC software, both for determination of Cq (2nd derivative method) and for melting curve analysis. Amplification efficiency was calculated using a linear regression method. All assays were inspected for distinct melting curves and the Tm was confirmed to be within known specifications for the assay. Assays returning 3 Cq less than the negative control and Cq<37 were accepted and sample runs not matching these criteria were omitted from further analysis (e.g., miR-92b-3p).

The stability values of candidate normalizers were assessed using the 'NormFinder' software[12]. Any qPCR data was normalized to the average Cp of internal normalizers detected in all samples (delta Cp; dCp=normalizer Cp–assay Cp). All miRNA analyses were conducted blind to the clinical data.

Cytokine and inflammatory biomarker measurements

Cytokine levels (IL-6, IL-8, IL-1β) were measured on a Luminex LX200 using Invitrogen's Human Inflammatory 5-Plex panel (Invitrogen/Life Technologies, Darmstadt, Germany) and Millipore filter plates (VWR Darmstadt) as per manufacturers' instructions. PCT was measured on a Kryptor instrument (Brahms, Henningsdorf, Germany). Levels of sCD25 were measured on commercially available microplate assays (Human IL-2 sRa (sCD25) OptEIA Set, Becton

Dickenson, San Diego, CA). All biomarker analyses were conducted blind to the clinical data as previously shown[13].

Statistical analyses

Unless specified, datasets were analyzed and plotted (including receiver operator curves, ROC) using the GraphPad Prism 6 and/or IBM SPSS Statistics 22 software. The D'Agostino and Pearson omnibus and/or Shapiro-Wilk tests were used to test normal data distribution. If not normally distributed, medians with interquartile ranges (IQR, rather than means and standard deviation, SD) are shown and Mann-Whitney U Test (rather than t-) tests were used to calculate p-values in 2-group comparisons. Significances across more than 2 groups were assessed by ANOVA (Kruskal-Wallis test). For the qPCR miRNA array dataset, a multiple testing correction was used to adjust ordinary p-values in order to control for the number of false positives (Benjamini-Hochberg adjusted p-values[14]). The CIR-miRNA score was generated as a linear combination of the top performing 6 miRNA measurements in severe sepsis and SIRS patients (n=40) and interpolated using IBM SPSS Statistics 22 by binary logistic regression to predict SIRS vs sepsis. In particular, the CIR-miRNA score (S) was mathematically defined as: $S = a_1x_1 + a_2x_2 + a_3x_3 + a_4x_4 + a_5x_5 + a_6x_6 + k$ where $x_{1\text{-}6}$ are the measurements of the top 6 miRNAs in a specific individual and the variables, $a_{1\text{-}6}$, and the constant, k, are the coefficients returned by the binary logistic regression model. In mathematical terms, the CIR-miRNA score is the natural logarithm of the odds of having SIRS vs sepsis given the measurements of the 6 top miRNAs, that is $ODDS=e^S$. Correlations between the interpolated CIR-miRNA scores and plasma levels of inflammatory mediators were evaluated using the Spearman rho and significances of the correlations in GraphPad Prism 6. Prior to assessing if

the sepsis/SIRS predictive value of CIR-miRNAs is preserved after adjusting for confounding variables, we verified the normal distribution of miRNA levels (continuous variables) and used IBM SPSS Statistics 22 multiple regression and hierarchical multiple regression to assess whether the confounding variables (SOFA score, age, sex, patient survival outcome, and time of sample collection) affect CIR-miRNA levels. It was noted that despite SOFA significantly correlates with levels of (in decreasing order) miR-191-5p ($R=-0.437$, $p=0.002$), miR-26a-5p ($R=-0.335$, $p=0.016$) and miR-23a-3p ($R=-0.262$, $p=0.049$) and Age weakly correlates with miR-192-5p ($R=-0.295$, $p=0.031$), in the intervals analysed in this study (Table 1), such effects were weak and not significant when corrected for other confounding variables. We then used IBM SPSS Statistics 22 hierarchical binary logistic regression models to assess if the Sepsis/SIRS predictive value is preserved after adjusting for the same confounding variables. In SPSS, SOFA score, age, sex, patient survival outcome, and time of sample collection (confounding variables) were force-entered in one block at step 1 of the regression models. Thereafter, in block 2 individual miRNAs or the top 6 CIR-miRNAs combined in one block were entered. In every case, step 2 significance was $p<0.05$ and the -2 Log likelihood improved over previous steps and the final model significance is shown in Supplementary Fig.4. The model coefficients from the “variables in the equation” SPSS output were then used to build the regression mathematical equations relative to each model in a similar manner as described above.

7. Supplemental Materials and Methods References

1 Azzouzi, I., Schmugge, M. & Speer, O. MicroRNAs as components of regulatory networks controlling erythropoiesis. *Eur. J. Haematol.* **89**, 1-9; DOI:10.1111/j.1600-0609.2012.01774.x (2012).

2 Pritchard, C. C. *et al.* Blood cell origin of circulating microRNAs: a cautionary note for cancer biomarker studies. *Cancer Prev. Res. (Phila.)* **5**, 492-497; DOI:10.1158/1940-6207.CAPR-11-0370 (2012).

3 Kirschner, M. B. *et al.* The Impact of Hemolysis on Cell-Free microRNA Biomarkers. *Frontiers in genetics* **4**, 94; DOI:10.3389/fgene.2013.00094 (2013).

4 Kirschner, M. B. *et al.* Haemolysis during sample preparation alters microRNA content of plasma. *PLoS One* **6**, e24145; DOI:10.1371/journal.pone.0024145 (2011).

5 Han, V., Serrano, K. & Devine, D. V. A comparative study of common techniques used to measure haemolysis in stored red cell concentrates. *Vox Sang.* **98**, 116-123; DOI:10.1111/j.1423-0410.2009.01249.x (2010).

6 Harboe, M. A method for determination of hemoglobin in plasma by near-ultraviolet spectrophotometry. *Scand. J. Clin. Lab. Invest.* **11**, 66-70; DOI:10.3109/00365515909060410 (1959).

7 Adamzik, M. *et al.* Free hemoglobin concentration in severe sepsis: methods of measurement and prediction of outcome. *Crit. Care* **16**, R125; DOI:10.1186/cc11425 (2012).

8 Lippi, G., Salvagno, G. L., Montagnana, M., Brocco, G. & Guidi, G. C. Influence of hemolysis on routine clinical chemistry testing. *Clin. Chem. Lab. Med.* **44**, 311-316; DOI:10.1515/CCLM.2006.054 (2006).

9 Oshiro, I., Takenaka, T. & Maeda, J. New method for hemoglobin determination by using sodium lauryl sulfate (SLS). *Clin. Biochem.* **15**, 83-88; DOI:10.1016/S0009-9120(82)91069-4 (1982).

10 Blondal, T. *et al.* Assessing sample and miRNA profile quality in serum and plasma or other biofluids. *Methods* **59**, S1-6; DOI:10.1016/j.ymeth.2012.09.015 (2013).

11 Jones, C. I. *et al.* Identification of circulating microRNAs as diagnostic biomarkers for use in multiple myeloma. *Br. J. Cancer* **107**, 1987-1996; DOI:10.1038/bjc.2012.525 (2012).

12 Andersen, C. L., Jensen, J. L. & Orntoft, T. F. Normalization of real-time quantitative reverse transcription-PCR data: a model-based variance estimation approach to identify genes suited for normalization, applied to bladder and colon cancer data sets. *Cancer Res.* **64**, 5245-5250; DOI:10.1158/0008-5472.CAN-04-0496 (2004).

13 Llewelyn, M. J. *et al.* Sepsis biomarkers in unselected patients on admission to intensive or high-dependency care. *Crit. Care* **17**, R60; DOI:10.1186/cc12588 (2013).

14 Benjamini, Y. & Hochberg, Y. Controlling the False Discovery Rate: A Practical and Powerful Approach to Multiple Testing. *J R Stat Soc Series B Stat Methodol* **57**, 289-300; DOI:10.2307/2346101 (1995).
